# Characteristics of A-type voltage-gated K^+^ currents expressed on sour-sensing type III taste receptor cells in mice

**DOI:** 10.1007/s00441-024-03887-6

**Published:** 2024-03-16

**Authors:** Takeru Moribayashi, Yoshiki Nakao, Yoshitaka Ohtubo

**Affiliations:** Graduate School of Life Science and Systems Engineering, Kyushu Institute of Technology, Hibikino 2-4, Kitakyushu, 808-0196 Japan

**Keywords:** In-situ whole-cell patch clamping, Voltage-gated potassium currents, Fungiform taste buds, Sour-sensing cells, Excitability

## Abstract

Sour taste is detected by type III taste receptor cells that generate membrane depolarization with action potentials in response to HCl applied to the apical membranes. The shape of action potentials in type III cells exhibits larger afterhyperpolarization due to activation of transient A-type voltage-gated K^+^ currents. Although action potentials play an important role in neurotransmitter release, the electrophysiological features of A-type K^+^ currents in taste buds remain unclear. Here, we examined the electrophysiological properties of A-type K^+^ currents in mouse fungiform taste bud cells using in-situ whole-cell patch clamping. Type III cells were identified with SNAP-25 immunoreactivity and/or electrophysiological features of voltage-gated currents. Type III cells expressed A-type K^+^ currents which were completely inhibited by 10 mM TEA, whereas IP_3_R3-immunoreactive type II cells did not. The half-maximal activation and steady-state inactivation of A-type K^+^ currents were 17.9 ± 4.5 (n = 17) and − 11.0 ± 5.7 (n = 17) mV, respectively, which are similar to the features of Kv3.3 and Kv3.4 channels (transient and high voltage-activated K^+^ channels). The recovery from inactivation was well fitted with a double exponential equation; the fast and slow time constants were 6.4 ± 0.6 ms and 0.76 ± 0.26 s (n = 6), respectively. RT-PCR experiments suggest that Kv3.3 and Kv3.4 mRNAs were detected at the taste bud level, but not at single-cell levels. As the phosphorylation of Kv3.3 and Kv3.4 channels generally leads to the modulation of cell excitability, neuromodulator-mediated A-type K^+^ channel phosphorylation likely affects the signal transduction of taste.

## Introduction

A single taste bud in mice contains 10–100 taste bud cells (TBCs) (Ogata and Ohtubo 2020; Ohtubo and Yoshii 2011), in which elongated TBCs are categorized into three cell types (types I–III) based on their morphology and function (Chaudhari and Roper 2010; Murray 1973; Roper 2013). Type I cells possibly function as glia-like supporting cells and express nucleoside triphosphate dephosphohydrolase-2 (NTPD2) and glutamate-aspartate transporter (Bartel et al. 2006; Lawton et al. 2000). Type II cells are taste receptor cells that express G protein-coupled taste receptors for sweet, bitter, and umami taste substances (Adler et al. 2000; Chandrashekar et al. 2000; Nelson et al. 2002; Nelson et al. 2001). They also express transient receptor potential melastatin 5 (Perez et al. 2002; Zhang et al. 2007), tetraethylammonium (TEA) and Cs^+^-insensitive outward rectifying (Kimura et al. 2014; Takeuchi et al. 2021), voltage-gated sodium Nav1.3 (Ohtubo 2021), calcium homeostasis modulator 1 and 3 (CALHM1/3) channels (Ma et al. 2018; Taruno et al. 2013), and among others. Contrarily, type III cells express ionotropic taste receptors such as otopetrin-1 (Otop1) for sour taste substances (Huang et al. 2008; Teng et al. 2019; Tu et al. 2018; Zhang et al. 2019) as well as voltage-gated Ca^2+^ (Clapp et al. 2006; DeFazio et al. 2006), TEA- and Cs^+^-sensitive voltage-gated K^+^ (Kimura et al. 2014; Takeuchi et al. 2021; Ye et al. 2016), and voltage-gated Nav1.3 channels (Ohtubo 2021). Taste receptor cells reportedly generated oscillating membrane depolarization with action potentials in response to sweet, bitter, and umami taste substances, whereas a sour tastant, 10-mM HCl, induced membrane depolarization with action potentials in type III cells (Nakao et al. 2022). As mentioned previously, it is likely that clarifying the cell-type-dependent expression of ion channels including the contribution of action potential generation promotes a better understanding of signal transduction of taste.

Inactivating voltage-gated K^+^ currents called “A-type” currents have characteristics of rapid activating and inactivating currents that play a role in the repolarization of action potentials in neurons (Belluzzi et al. 1985; Gao and Ziskind-Conhaim 1998; Storm 1987). In mammals, the following six types of genes encode A-type voltage-gated K^+^ channels (Kv channels): Kv1.4, Kv3.3, Kv3.4, Kv4.1, Kv4.2, and Kv4.3 (Covarrubias et al. 1994; Fernandez et al. 2003; Franqueza et al. 1999; Hashimoto et al. 2000; Petersen and Nerbonne 1999; Zemel et al. 2018). Additionally, an erg3 current through Kv11.3 channel has a predominant transient component that decayed to a sustained plateau (Mauerhofer and Bauer 2016; Shi et al. 1997). Although TBCs functionally express rapid activating and inactivating voltage-gated K^+^ channels, there is a paucity of knowledge on the electrophysiological features of A-type K^+^ currents among respective cell types and gene expression among TBCs.

In the present study, we aimed to examine the electrophysiological features of A-type K^+^ channels using in-situ whole-cell clamping, immunohistostaining to identify cell types, and reverse transcription polymerase chain reaction (RT-PCR) techniques. We showed that sour taste-responding type III taste receptor cells functionally expressed A-type K^+^ currents, whereas type II taste receptor cells did not. The electrophysiological properties of A-type K^+^ currents in type III cells were analogs to those of Kv3.3 and Kv3.4 channels. RT-PCR experiments showed that Kv3.3 mRNA was observed in all seven individual experiments. Single-cell RT-PCR experiments indicated that Kv3.3 and Kv3.4 mRNAs remained undetected at the single-cell level but could be detected at the taste bud level. Our findings suggest that transient and high voltage-activated K^+^ channels, probably Kv3.3 and Kv3.4 channels, are principal components of A-type K^+^ currents in type III fungiform TBCs. The physiological roles of A-type K^+^ channels in taste transduction were made for discussion.

## Materials and methods

### Preparation of peeled epithelia

Mouse lingual epithelia were prepared as described previously (Furue and Yoshii 1997, 1998; Ohtubo et al. 2001). In brief, ddY-strain male mice (5–11 weeks old) were euthanized by CO_2_ suffocation and decapitation, hypodermically injected with a collagenase solution into their tongues, and then incubated at 25 °C for approximately 3 min; subsequently, the lingual epithelia were removed with forceps. The epithelia were mounted on a recording platform with the basolateral membrane side of TBCs (the opposite side of the tongue surface) facing upward and the apical membranes facing inside the recording platform. The recording platform was placed under a microscope equipped with a 60 × water-immersion objective (BX50, Olympus Corporation, Tokyo, Japan). The basolateral membrane was always irrigated with either a physiological saline or a stimulating solution containing blockers. The apical membrane was acclimated to the physiological saline. When sour taste recordings were performed, the apical membrane was irrigated with a deionized water or 10-mM HCl dissolved in the deionized water.

### Electrophysiological recordings

Conventional whole-cell voltage-clamp conditions were utilized according to previous studies (Nakao et al. 2020; Ohtubo 2021; Takeuchi et al. 2011). In brief, electrical responses were recorded with electrodes (ca. 5 MΩ) filled with a K-gluconate electrode solution containing 1-mg/ml biocytin (Sigma-Aldrich, MO, USA), amplified with a voltage-clamp amplifier (Axopatch 200B, Molecular Devices, San Jose, CA, USA), filtered at 10 kHz, digitized with an A/D converter (Digidata 1322A, Molecular Devices), and analyzed using pCLAMP data acquisition and analysis software (ver. 10, Molecular Devices). The membrane capacitance of TBCs was calculated from the current responses to a voltage step depolarization from −70 to −50 mV and measured using pCLAMP data acquisition and analysis software.

To enable efficient measurements from type III cells, taste bud cells were selected based on the characteristics of a family of voltage-gated currents, as reported in our previous studies (Iwamoto et al. 2020; Kimura et al. 2014; Nakao et al. 2022; Takeuchi et al. 2021). If the taste bud cells exhibited rapidly activating outwardly rectifying currents, almost no tail currents, and large transient inward currents (voltage-gated Na^+^ currents) characteristic of type III cells under conventional whole-cell voltage-clamp conditions, we continued the experiments and applied the voltage steps to activate A-type K^+^ currents. If the cells showed characteristics other than those of type III cells (e.g., slowly activating outwardly rectifying currents, large tail currents, and little or no voltage-gated Na^+^ and outwardly rectifying currents), we stopped the recordings and tried to record from other cells.

Perforated whole-cell current-clamp was performed to record sour taste responses using the K-gluconate electrode solution containing amphotericin B (Fujifilm Wako Pure Chemical Corporation, Osaka, Japan) and Pluronic^®^ F-127 (Sigma-Aldrich, MO, USA), as reported previously (Nakao et al. 2022). Briefly, the recording electrode with 2–4 MΩ resistance in physiological saline was placed on the basolateral membranes of TBCs. By applying negative pressure, we made the GΩ seal and then monitored the membrane capacitance by applying rectangular pulses from −70 mV to −65 mV continuously. When the membrane capacitance reached steady-state levels at approximately 10–20 min, we recorded a family of voltage-gated currents under a voltage-clamp mode. Subsequently, the mode was changed to the current-clamp mode, and the sour taste responses were examined. The resting membrane potentials of TBCs were controlled at approximately −60 mV by injecting the currents. The sour taste substance (10-mM HCl) was applied to only the apical membranes of the TBCs.

The amphotericin B stock solution at a concentration of 25–30 mg/ml, which was diluted with dimethylsulfoxide (DMSO), was sonicated, stored at −20 ℃, and used within 5 days. The Pluronic^®^ F-127 (20 mg/ml) solution dissolved in the K-gluconate solution was sonicated. The K-gluconate electrode solution was prepared in the K-gluconate solution by adding an amphotericin B stock solution to the final concentration of 300–500 μg/ml and Pluronic^®^ F-127 solution to the final concentration of 250 μg/ml. Then, the K-gluconate electrode solution was sonicated in a light-shielding glass bottle for 1–3 min, and filtered with 0.22-μm membrane-filter unit (MILLEX^®^-GP; Merck, Darmstadt, Germany). The K-gluconate electrode solution was stored in a container with ice, protected from the light just before use, and renewed every 4 h.

### Data analysis

The standard current-voltage (*I*–*V*) families were obtained using 150-ms pulses from a holding potential of −70 mV to a range of potentials (−100 mV to +80 mV in 5-mV increments) every 0.5-s to examine the whole-cell recording configuration. For statistical comparison, the standard *I*–*V* families were recorded to compare the activation kinetics of outwardly rectifying currents at +50 mV, as a characteristic of type III cells. The activation time constant at +50 mV was obtained using a single exponential curve fitting to the current trace from the beginning to the peak current amplitude using the following equation:

$$I(t)=A\lbrack1-\exp\;((-t+d)/\tau)\rbrack$$

where *I*_(t)_ is the flow of outwardly rectifying currents at time (t), *A* is the current amplitude at infinite time, *d* is the delay required to obtain an adequate fit to a single exponential function, and *τ* is the time constant.

For the analysis of the activation of A-type voltage-gated K^+^ currents, activation and inactivation pulses were applied under the whole-cell voltage-clamp conditions. The activation pulse protocol comprised 1-s conditioning pulses from −70 mV holding potentials to −90 mV, followed by a test pulse from −40 mV to +60 mV in 5-mV increments for 0.8-s. The inactivation pulse protocol comprised 1-s conditioning pulses from −70 mV holding potentials to 0 mV, followed by a test pulse from −40 mV to +60 mV in 5-mV increments for 0.8-s. By subtracting the current trace induced by the activation pulse protocol to that by the inactivation pulse protocol, the activation of A-type voltage-gated K^+^ currents was obtained. The peak currents, thus obtained, were normalized to that at +50 mV, and plotted against the membrane voltage potentials. Data were fitted with the following Boltzmann equation:

$$I\scriptsize K\normalsize=1/\lbrack1+\exp((Va\scriptsize1/2\normalsize-V)/s)\rbrack$$

where *I*_K_ is the relative K^+^ currents, *Va*_1/2_ is the half-maximal voltage of activation, and *s* is the slope factor.

Steady-state inactivation was determined using a double pulse protocol consisting of 2-s conditioning pulses to command potentials between −50 mV and +40 mV in 10-mV steps, followed by a constant test pulse of +60 mV for 1.5-s. The pulse interval was 4 s. The amplitude of peak K^+^ currents during the test pulses was normalized to the peak current at −40 mV, and plotted as function of the conditioning potentials. The data were fitted using the following Boltzmann equation:

$$I\scriptsize K\normalsize = 1/[1 + \exp((V - Vi\scriptsize1/2\normalsize)/s)]$$

where *I*_K_ is the normalized K^+^ current, *Vi*_1/2_ is the half-maximal voltage of inactivation, and *s* is the slope factor.

Recovery from inactivation was evaluated using a double pulse protocol consisting of an initial control pulse (*I*_*cont*_) at +60 mV for 1-s, a variable recovery interval at −70 mV, and a test pulse (*I*_*test*_) at +60 mV for 1-s. The control pulse interval was 8s. The peak current ratio *I*_*test*_/*I*_*cont*_ was calculated and plotted against the interval time (Δ*t*). To obtain the time constant of the recovery from inactivation, data were fitted using the following double exponential function:

$$I\scriptsize test\normalsize/I\scriptsize cont\normalsize = A \times \exp(- \Delta t/\tau 1) + (1 - A) \times \exp(- \Delta t/\tau2)$$

where *A* is the fraction of the total A-type K^+^ current described by a fast time constant (*τ1*) and *τ2* represents a slow time constant.

The inactivation process of the A-type K^+^ currents were fitted by using the following equation:

$$I(t)=\lbrack A\scriptsize 1\normalsize (\exp\;((-t/\tau1))\rbrack+\lbrack A\scriptsize 2\normalsize(\exp((-t/\tau2))\rbrack+I\scriptsize 0$$

where *I*_(t)_ is the total A-type K^+^ current at time *t*. *A*_*1*_ and* A*_*2*_ are the peak magnitudes of the two components related to time constants *τ1* and *τ2*. *I*_0_ is the non-inactivating residual current.

### Immunohistochemistry

Cell types electrophysiologically investigated were identified immunohistochemically as described previously(Mori et al. 2016; Ohtubo 2021; Ohtubo and Yoshii 2011). Briefly, the epithelia containing a biocytin-injected TBC were fixed immediately after the electrophysiological recording with a fixation solution overnight or for up to 24 days, washed six times for 10 min with phosphate-buffered saline (PBS), and incubated in 10-mM citrate buffer (pH 6.0) for 20 min at 85 °C. After briefly washing with PBS, the epithelia were then incubated in a blocking solution for 4 h. The fixed epithelia were incubated for 24–48 h at 4 °C with a mixture of the following primary antibodies: anti-type III inositol 1,4,5-triphoshate receptor (IP_3_R3) mouse monoclonal antibody (RRID; AB_397705, dilution; 1:50, BD, Franklin Lakes, NJ, USA), and anti-synaptosomal-associated protein 25 (SNAP-25) rabbit polyclonal antibody (AB_261576, 1:500, Sigma, St. Louis, MO, USA). The antibodies were diluted with the blocking solution to the concentration indicated.

Following incubation with primary antibodies, the epithelia were washed in PBS six times for 10 min at 25 °C and incubated for 24–48 h at 4 °C with a mixture containing fluorescently-labeled secondary antibodies and streptavidin: Alexa Fluor 488-labeled donkey anti-rabbit IgG (AB_141708, 1:400, Thermo Fisher Scientific, Waltham, MA, USA), Alexa Fluor 555-labeld donkey anti-mouse IgG (AB_2536180, 1:400, Thermo Fisher Scientific), and Alexa Fluor 633-conjugated streptavidin (AB_2313500, 1:100, Thermo Fisher Scientific). After incubation with secondary antibodies, the epithelia were washed six times for 10 min in PBS, mounted using glycerol, and coverslipped. The stained epithelia were viewed using an epifluorescence microscope (BX-URA2, Olympus Corporation) equipped with a CCD camera (DS-Qi1Mc, Nikon Corporation, Tokyo, Japan). Fluorescent images were captured and analyzed using NIS-Elements (Nikon Corporation).

#### RT-PCR

Fungiform taste buds in the peeled epithelia were collected using a suction micropipette as described previously(Hayato et al. 2007; Iwamoto et al. 2020; Ohtubo 2021). Briefly, the epithelia were peeled off and mounted on the recording platform, and the basolateral membrane surface of taste buds was treated with a Ca^2+^ and Mg^2+^ nominally-free solution for 60–90 s, and then placed under a microscope. Each taste bud was liberated by puffing the Ca^2+^ and Mg^2+^ nominally free solution onto the cleft between a taste bud and epithelium cells, surrounding it with the physiological saline. Then, another micropipette was used to suck up the taste bud. The liberated taste buds were transferred into Isogen™ (NIPPON GENE, Tokyo, Japan), homogenized, and stored at −80 °C.

Total mRNA was extracted as a water soluble fraction, precipitated in 37.5% isopropanol supplemented with Dr.GenTLE™ precipitation carrier (Takara BIO, Tokyo, Japan), washed by ethanol precipitation, and then treated with DNase (Takara BIO) following the manufacturer’s protocol. Reverse transcription (RT) was performed at 55 °C for 30 min. PCR cycles consisted of an initial step of 94 °C for 15 min and 45 subsequent cycles of 94 °C for 30 s (denaturation), 58 °C for 1 min (annealing), 72 °C for 3 min (extension), and a final extension step of 72 °C for 10 min in a thermal cycler (ASTEC PC-808, Fukuoka, Japan). PCR products were analyzed by 2% agarose gel electrophoresis, stained with ethidium bromide (0.5 μg/ml), and visualized by UV illumination. The RT-PCR test was performed with a QIAGEN-One-step-RT-PCR kit (QIAGEN GmbH, Hilden, Germany) per the manufacturer’s instructions.

The mouse whole brains were immediately homogenized in Isogen™ and stored at −80 °C, and used as positive controls for each primer set (Table 1). Annealing temperatures of 58 °C yielded a clear single band of the correct size on agarose gels for each primer set. The amplification of housekeeping gene β-actin and cell-type markers of nucleoside triphosphate diphosphohydrolase-2, phospholipase C β2, and SNAP-25 was used as a positive control.

**Table 1:** Sequences of primer sets used for RT-PCR and single-cell RT-PCR experiments

Gene (MGI symbol)	Forward primer 5' - 3'	Reverse primer 5' - 3'	Product size (bp)
Kv1.4 (Kcna4)	TCCAGTAACGAGGACTCTGC	GCCTTCTACTGATGGCTTGA	517
Kv3.3 (Kcnc3)	TTTTGAGGACCCCTACTCGT	CCACTTCCAAGTAGAAGGGC	353
outer	GGACGAGTTCTTCTTCGACC	CTTTGGAGCTGAGACCTGAC	815
Kv3.4 (Kcnc4)	AGCTCACCTTCTGGGGTATC	TGACAAAGTCCAGCGTATCA	546
outer	AGATCATCATCAACGTGGGC	ACAAACCACTCAATCCCACC	906
Kv4.1 (Kcnd1)	CAGCCGCAGTACCTCAGTAT	TTGACAGTCTCAGGGAGGAG	405
Kv4.2 (Kcnd2)	AAAAGACTTTCCGCATTCCT	GACGATATTTCCTCCCGAAT	272
Kv4.3 (Kcnd3)	CCTGAAATCAAGGCAAAGAA	ACTCCTTCGTGTCCTCATTG	342
Kv11.3 (Kcnh7)	GAGCAGATGTCTTGCCAGAA	TCCAGAACTCGAGTCACTGT	680
NTPD2 (Entpd2)	GTGACTGCCAACTACCTGCT	GACCCATAGTGCATGGAGAC	352
outer	CCTCAAGTATGGCATCGTTC	TATTGAAGAGCCCAGAGACG	848
PLCβ2 (Plcb2)	CTCGCTTTGGGAAGTTTGC	GCATTGACTGTCATCGGGT	226
outer	AGCCTAAGCTTCCTCTCCTG	AGGAGTTGAGTCGAGGGTCT	865
SNAP25 (Snap25)	GGCAATAATCAGGATGGAGTAG	AAATTTAACCACTTCCCAGCA	310
outer	AGG AAG GGA TGG ACC AAA TC	GGG GGT GAC TGA CTC TGT GT	600
β–actin (Actb)	GTAAAGACCTCTATGCCAACAC	GTGTAAAACGCAGCTCAGTAAC	289

Outer primers were used for multiplex nested single-cell RT-PCR

Primer sets for Kv3.3, Kv3.4, and cell-type markers in the RT-PCR experiments were employed as inner primers for the single-cell RT-PCR

### Single-cell RT-PCR

A single TBC from the peeled epithelia was collected using a suction micropipette, as described previously (Ohtubo 2021). Briefly, the epithelia were peeled off and mounted on the recording platform. Then, the basolateral membrane surface of taste buds was treated with a Ca^2+^ and Mg^2+^ nominally free solution for 90–120 s and placed under a microscope. The tip of the suction micropipettes was located on the surface of a TBC, the negative presser was applied to the micropipettes, and the TBC was pulled out of the taste bud. Then, another micropipette with a large orifice was used to suck up the TBC. The single TBC was placed in a tube, where RT and first-round amplification were conducted using the QIAGEN-One-step-RT-PCR kit, according to the manufacturer’s instructions.

A 50-μl reaction mixture containing 0.5 μl of RNase inhibitor (Takara BIO) was prepared according to the manufacturer’s instructions. After the RT reaction at 50 °C for 30 min, the first round of PCR was subsequently performed in the same tube with a 15-min preincubation at 95 °C, followed by 35 cycles of denaturation (at 94 °C for 30 s), annealing (at 58 °C for 60 s), and amplification (at 72 °C for 90 s) in a thermal cycler (ASTEC GeneAtlas G02, Fukuoka, Japan). The denaturation and amplification temperatures were chosen following the kit manual, and the annealing temperature was determined experimentally both in this and the second round of PCR described below.

First-round PCR products were re-amplified for 40 cycles (94 °C, 30 s; 58 °C, 30 s; 68 °C, 60 s) in separate reactions using the inside primer pairs for each template. Each 20 μl second-round reaction mix contained the following: 0.5 μl of KOD-plus- polymerase (TOYOBO, Osaka, Japan), 2 μl of 10X PCR buffer, 0.8 μl of 25 mM MgSO_4_, 2 μl of 2 mM dNTP mix, 0.6 μM of each inside primer pair (Table 1), and 2 μl of first-round PCR products. After the second-round amplification, the PCR products were analyzed as described in the RT-PCR section above.

### Solutions

All solutions were prepared with deionized water. The K-gluconate electrode solution comprised 120-mM K-gluconate, 2.4-mM CaCl_2_, 0.5-mM MgCl_2_, 10-mM EGTA, 30-mM KOH, 5-mM Na_2_ATP, 0.3-mM Na_3_GTP, 10-mM HEPES, 1-mg/ml biocytin, and KOH to pH 7.2. Physiological saline was prepared as follows: 150-mM NaCl, 5-mM KCl, 2-mM CaCl_2_, 0.5-mM MgCl_2_, 10-mM glucose, 5-mM HEPES, and NaOH to pH 7.4. The collagenase solution contained 4 mg/ml collagenase (collagenase type I, Fujifilm Wako Chemicals, Tokyo, Japan) dissolved in physiological saline. The Ca^2+^and Mg^2+^ nominally free solution was composed of 140-mM NaCl, 5-mM KCl, 2-mM EGTA, 10-mM glucose, 10-mM HEPES, and NaOH to pH 7.4. The PBS solution consisted of 137-mM NaCl, 2.67-mM KCl, 8.09-mM Na_2_HPO_4_, and 1.47-mM KH_2_PO_4_. The fixation solution contained fresh 4% paraformaldehyde in PBS. The blocking solution contained 3% normal donkey serum dissolved in PBS, supplemented with 1% bovine serum albumin and 0.3% Triton X-100.

### Statistical analysis

By using immunohistostaining, two type II cells immunoreactive to IP_3_R3 and eight type III cells immunoreactive to SNAP-25 were identified after the patch clamp recordings. Moreover, based on the electrophysiological features of voltage-gated currents, 10 type III cells were identified. The total numbers of TBCs identified with immunoreactivity and/or electrophysiological features were as follows: two type II cells and 18 type III cells. The electrophysiological features between immunohistochemically and electrophysiologically identified type III cells were analyzed using unpaired t-tests. *P*-values < 0.05 were considered statistically significant. Data are shown as means ± standard deviation (SD) unless noted otherwise.

## Results

### Sour taste-responding TBCs generate action potentials with afterhyperpolarization

**Fig. 1:**
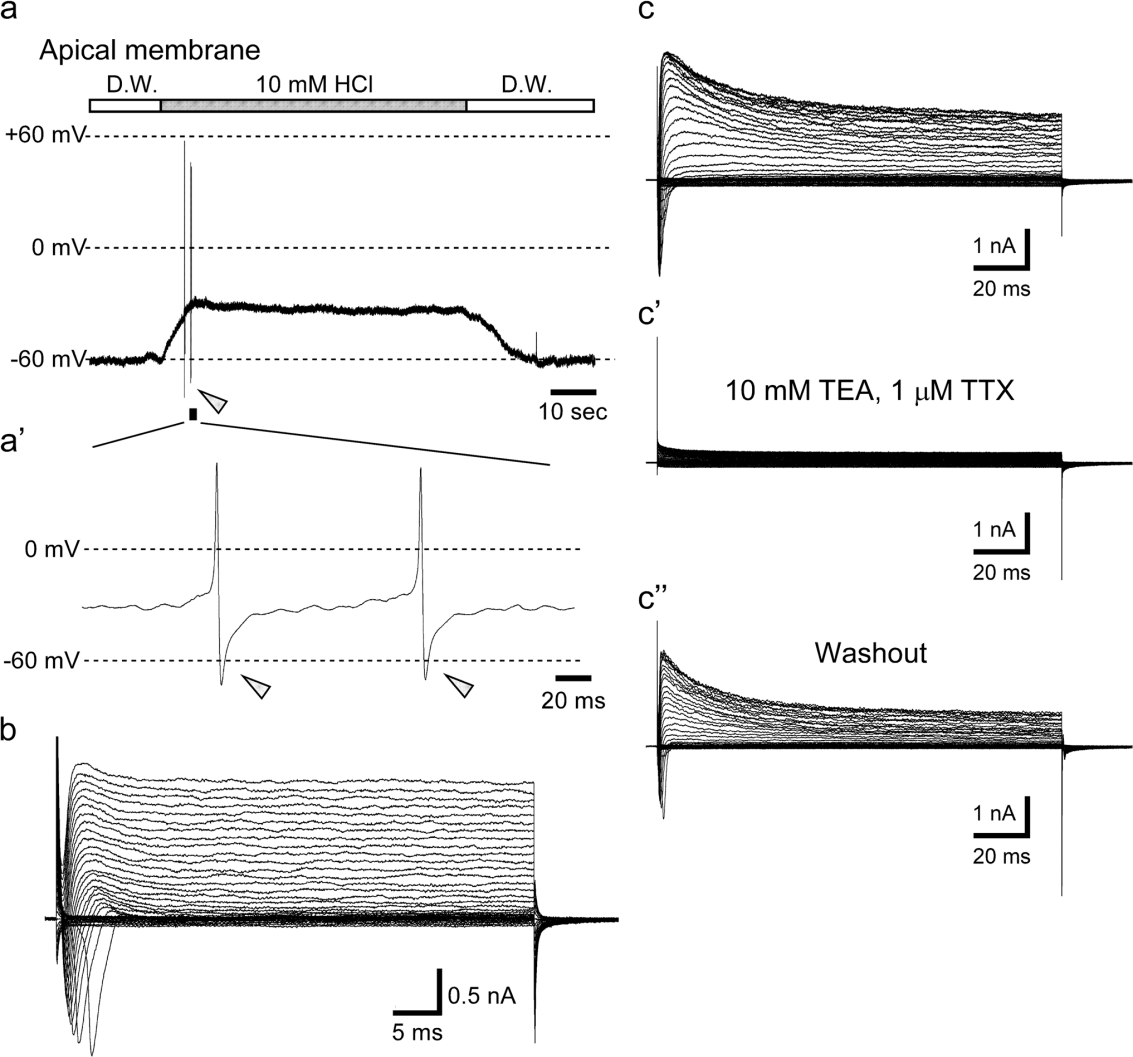
Sour stimuli to the apical membranes on TBCs induce membrane depolarization with action potentials that exhibited afterhyperpolarization. A sour taste substance (10-mM HCl) that was applied only to the apical membrane of TBCs elicited the membrane depolarization with action potentials which were observed at the rising phase of depolarization (**a**). These action potentials showed afterhyperpolarization potentials (arrowheads) that reached close to the equilibrium potentials of K^+^ channels (**a’**). A family of voltage-gated currents (**b**) obtained from sour-responding taste receptor cells shown in (**a**). This TBC exhibited the voltage-gated transient inward currents and rapid activated, voltage-gated outward currents. Note that the current-clamp (**a**) and voltage-clamp (**b**) recordings were performed under the perforated whole-cell patch conditions. Representative voltage-gated currents obtained from the conventional whole-cell voltage clamping (**c**). The voltage-gated transient inward currents and rapid activated, voltage-gated outward currents were completely inhibited by 1-μM TTX and 10-mM TEA, respectively, which were applied to the basolateral membrane (**c’**). Both voltage-gated currents in magnitude were recovered by washout (**c’’**)

When a sour taste substance, i.e.,10-mM HCl, was applied only to the apical membranes of the TBCs, a TBC generated the membrane depolarization with action potentials that were observed at only the rising phase of depolarization (Fig. 1a–a’), in agreement with a previous study (Nakao et al. 2022; Ohtubo et al. 2001). The action potentials were reached to approximately +60 mV close to the equilibrium potentials of voltage-gated Na^+^ channels. Then, the membrane potentials promptly and transiently hyperpolarized to approximately −70 mV close to the equilibrium potentials of voltage-gated K^+^ channels (Fig. 1a–a’). A family of voltage-gated currents obtained from the TBC responding to the sour stimulus showed transient inward currents and rapid activating and inactivating outwardly rectifying currents (Fig. 1b). As the expression of rapid activated outwardly rectifying currents was an electrophysiological feature of type III cells (Iwamoto et al. 2020; Nakao et al. 2022; Takeuchi et al. 2011), the sensitivity of TEA was investigated under conventional voltage clamping. When 10-mM TEA was applied to the basolateral membrane of TBCs, the outwardly rectifying currents were completely and reversibly inhibited (Fig. 1c–c’’). These data suggest that type III cells express rapid activating and inactivating voltage-gated K^+^ channels, consistent with the findings of previous studies (Iwamoto et al. 2020; Kimura et al. 2014; Takeuchi et al. 2021).

### Expression of transient A-type voltage-gated K^+^ currents on type III cells

**Fig. 2:**
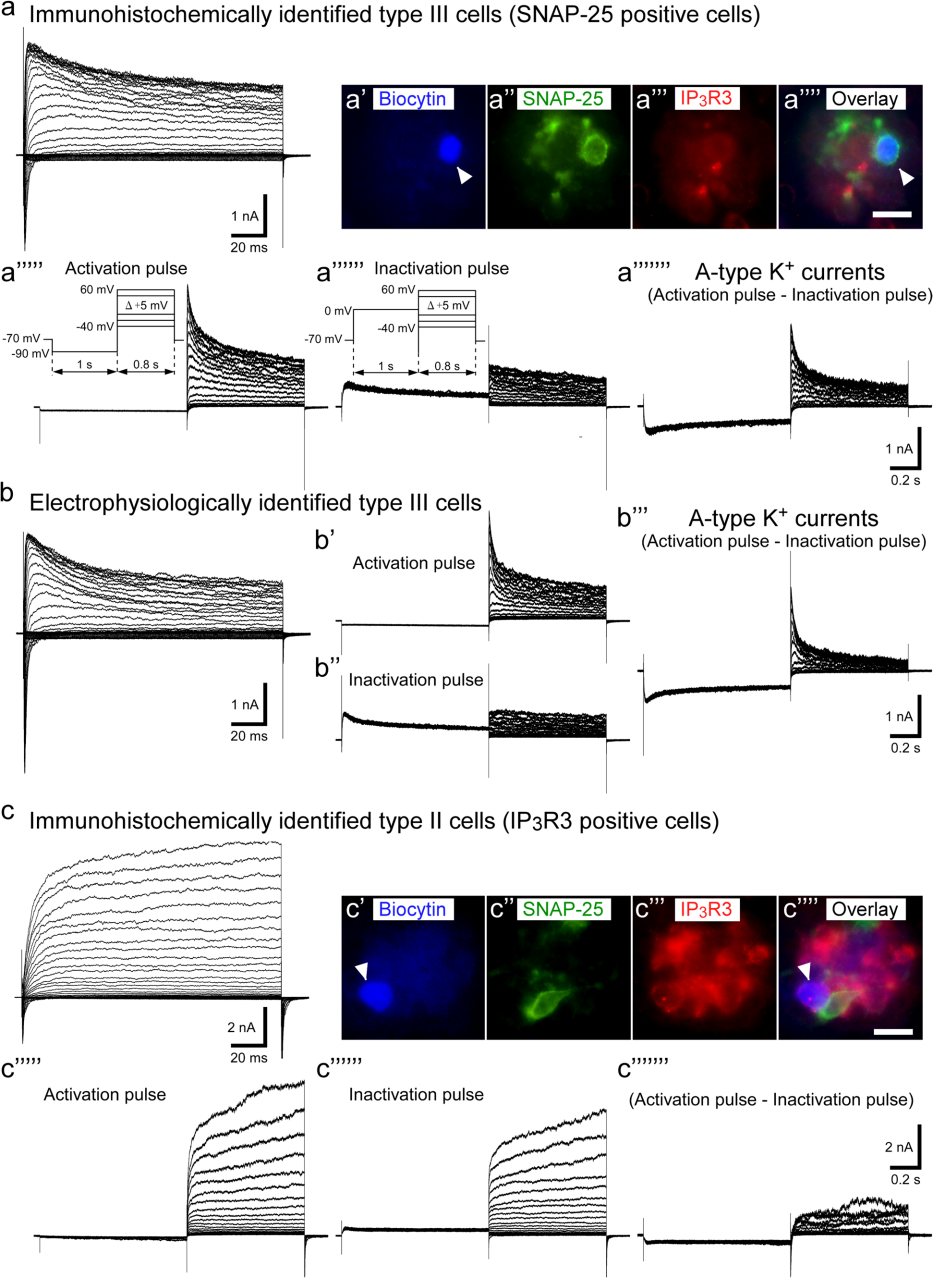
Type III cells generate transient A-type voltage-gated K^+^ currents, whereas type II cells did not. A family of voltage-gated currents (**a**, upper left) obtained from immunohistochemically identified type III cells. This biocytin-injected TBC (arrowhead) was immunoreactive to SNAP-25 (**a’**–**a’’’’**). Representative traces of A-type K^+^ currents on SNAP-25 immunoreactive TBCs **(a’’’’’**–**a’’’’’’’**). A-type K^+^ currents (**a’’’’’’’**) were obtained by subtracting the current traces induced by the activation pulse (**a’’’’’**) from those by the inactivation pulse (**a’’’’’’**). A family of voltage-gated currents obtained from electrophysiologically identified type III cells (**b**). The outwardly rectifying currents exhibited rapid activating currents that were a characteristic of type III cells. Furthermore, electrophysiologically identified type III cells generated A-type K^+^ currents (**b’’’**). A family of voltage-gated currents (**c**) obtained from immunohistochemically identified type II cells. This biocytin-injected TBC (arrowhead) was immunoreactive to IP_3_R3 (**c’**–**c’’’’**). Type II cells did not generate A-type K^+^ currents (**c’’’’’’’**) that were obtained by subtracting the current traces (**c’’’’’** and **c’’’’’’**)

For further detailed analysis of the rapid activating and inactivating voltage-gated K^+^ channels, we applied electrical pulse protocols for activating transient A-type voltage-gated K^+^ currents to TBCs; then, the cell type of TBC-recorded voltage-gated currents was identified by immunoreactivity to SNAP-25, a type III cell maker, and IP_3_R3, a type II cell marker, antibodies. A-type voltage-gated K^+^ currents were obtained by subtracting the currents induced by the inactivation pulse protocol from the currents induced by the activation pulse protocol. SNAP-25-immunoreactive type III cells showed the expression of A-type voltage-gated K^+^ currents (n = 8, Fig. 2a–a’’’’’’’). During the immunohistostaining process, we lost approximately 50% of TBCs that recorded voltage-gated currents. In such cases, we identified their cell types based on the electrophysiological properties of voltage-gated ion channels, because type III cells express rapid activating TEA-sensitive K^+^ currents and a relatively large current magnitude of voltage-gated Na^+^ channels, compared type I cells as previously reported (Iwamoto et al. 2020; Kimura et al. 2014; Takeuchi et al. 2021). The activation time constants of outwardly rectifying currents at + 50 mV in SNAP-25-immunoreactive and electrophysiologically identified cells were 0.84 ± 0.11 (n = 8) and 0.90 ± 0.14 (n = 10) ms, respectively, showing no significant difference between them (*P* = 0.33, unpaired t-test).

Both immunohistochemically and electrophysiologically identified type III cells generated A-type voltage-gated K^+^ currents (Fig. 2a–a’’’’’’’ and 2b–b’’’). Contrarily, the immunohistochemically identified type II cells (IP_3_R3-immunoreactive cells) did not express A-type voltage-gated K^+^ currents (n = 2, Fig. 2c–c’’’’’’’). Additionally, previous studies have revealed that the main components of outwardly rectifying currents in type II cells include the slowly activating TEA- and Cs^+^-insensitive currents that can pass through CALHM1/3 channels and hemichannels (Iwamoto et al. 2020; Kimura et al. 2014; Takeuchi et al. 2021). In these previous studies, the activation time constant of outwardly rectifying currents in type II cells was approximately 6 ~ 20 ms at +50 mV, which was significantly slower than that in type III cells (~ 1 ms at +50 mV). In addition, the fraction of TEA- and Cs^+^-sensitive voltage-gated K^+^ currents in type II cells was low (~ 20% of total outwardly rectifying currents) compared with that in other cell types. It is likely that type II cells expressed no or little A-type K^+^ currents because the activation time constant of A-type K^+^ currents (Fig. 4a) was much faster than that of 6 ~ 20 ms in type II cells. Taken together, these results suggest that rapid activating A-type voltage-gated K^+^ currents are functionally expressed on type III taste cells.

### Voltage dependency of activation and steady-state inactivation

**Fig. 3:**
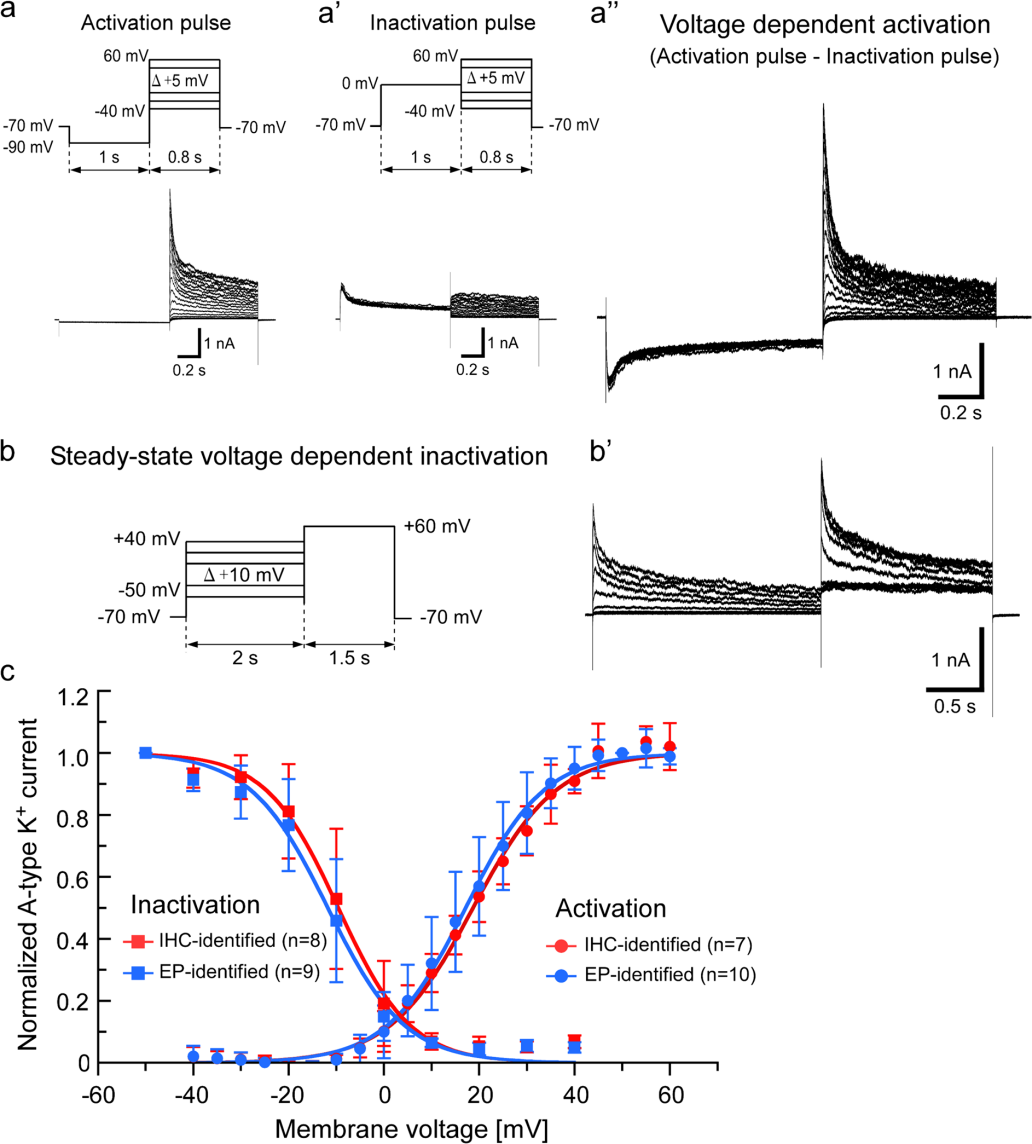
Activation and steady-state inactivation of A-type voltage-gated K^+^ current. Representative traces of activation of A-type K^+^ currents (**a’’**) that were obtained by subtracting the current trace induced by activation plus (**a**) from that by inactivation plus (**a’**). Representative traces of steady-state voltage-dependent inactivation of A-type K^+^ current (**b**–**b’**). A-type K^+^ currents for immunohistochemically (IHC)-identified and electrophysiologically (EP)-identified type III cells were normalized to the current elicited from a holding potential of + 50 mV for activation and of − 50 mV for inactivation (**c**). The respective current values were fitted with Boltzmann functions (lines)

Voltage-dependent activation of A-type voltage-gated K^+^ currents was obtained by subtracting the currents induced by the inactivation pulse protocol from the currents induced by the activation pulse protocol (Fig. 3a–a’’). The A-type K^+^ currents become apparent when the membrane potential was depolarized to potential more positive than approximately − 10 mV (Fig. 3c). The activation curves of normalized A-type K^+^ currents at +50 mV were fitted with a Boltzmann function. The half-maximal activation (*Va*_1/2_) of SNAP-25 immunoreactive and electrophysiologically identified type III cells was 18.8 ± 2.6 (n = 7) and 17.3 ± 5.4 (n = 10) mV, respectively, showing no significant difference (*P* = 0.52, unpaired t-test, Fig. 3c and Table 2). That is, the *Va*_1/2_ of A-type voltage-gated K^+^ currents in type III cells was 17.9 ± 4.5 mV (n = 17), which bears resemblance to the *Va*_1/2_ of Kv3.3 and Kv3.4 channels at ~ 7 and 13–19 mV, respectively, which are more depolarized values as compared to the other A-type K^+^ channels (Coetzee et al. 1999; Fernandez et al. 2003; Johnston 2021). Therefore, it suggests that type III cells express high voltage-activated A-type K^+^ channels.

**Table 2:** Electrophysiological properties of A-type voltage-gated K^+^ currents on type III cells of fungiform taste buds

	Activation		Inactivation		Recovery from inactivation		
	*Va*_1/2_ [mV]	*S* [mV/e-fold potential change]	*Vi*_1/2_ [mV]	*S* [mV/e-fold potential change]	*τ1* [ms]	*τ2* [s]	A (% of *τ1*)
Type III cells	17.9 ± 4.5 (17)	8.1 ± 1.3 (17)	− 11.0 ± 5.7 (17)	6.8 ± 1.1 (17)	6.4 ± 0.5 (6)	0.76 ± 0.26 (6)	58.9 ± 2.3 (6)
SNAP-25-IRCs	18.8 ± 2.6 (7)	8.7 ± 1.2 (7)	− 9.9 ± 6.0 (8)	6.6 ± 1.2 (8)	6.3 ± 0.5 (3)	0.90 ± 0.07 (3)	60.0 ± 1.8 (3)
Electro ICs	17.3 ± 5.4 (10)	7.7 ± 1.2 (10)	− 11.9 ± 5.6 (9)	7.0 ± 1.0 (9)	6.5 ± 0.5 (3)	0.62 ± 0.32 (3)	58.0 ± 2.4 (3)

The values represent means ± SD with the experiment number within parentheses

SNAP-25-IRCs, SNAP-25-immunoreactive cells; Electro ICs, electrophysiologically identified cells

Voltage dependency of steady-state inactivation was investigated by applying the electrical pulse protocol, as shown in Fig. 3b–b’. The half-maximal steady-state inactivation (*Vi*_1/2_) was − 9.9 ± 6.0 mV (n = 8) for SNAP-25 immunoreactive type III cells and − 11.9 ± 5.6 mV (n = 9) for electrophysiologically identified type III cells (Fig. 3c and Table 2), showing no significant difference between them. That is, the *Vi*_1/2_ of A-type voltage-gated K^+^ currents in type III cells was − 11.0 ± 5.7 mV (n = 17), which is relatively similar to the functional properties of Kv3.3 (−22 and −30 mV) (Fernandez et al. 2003) and Kv3.4 (−20 to −32 mV) (Coetzee et al. 1999) channels among the other A-type K^+^ channels.

**Fig. 4:**
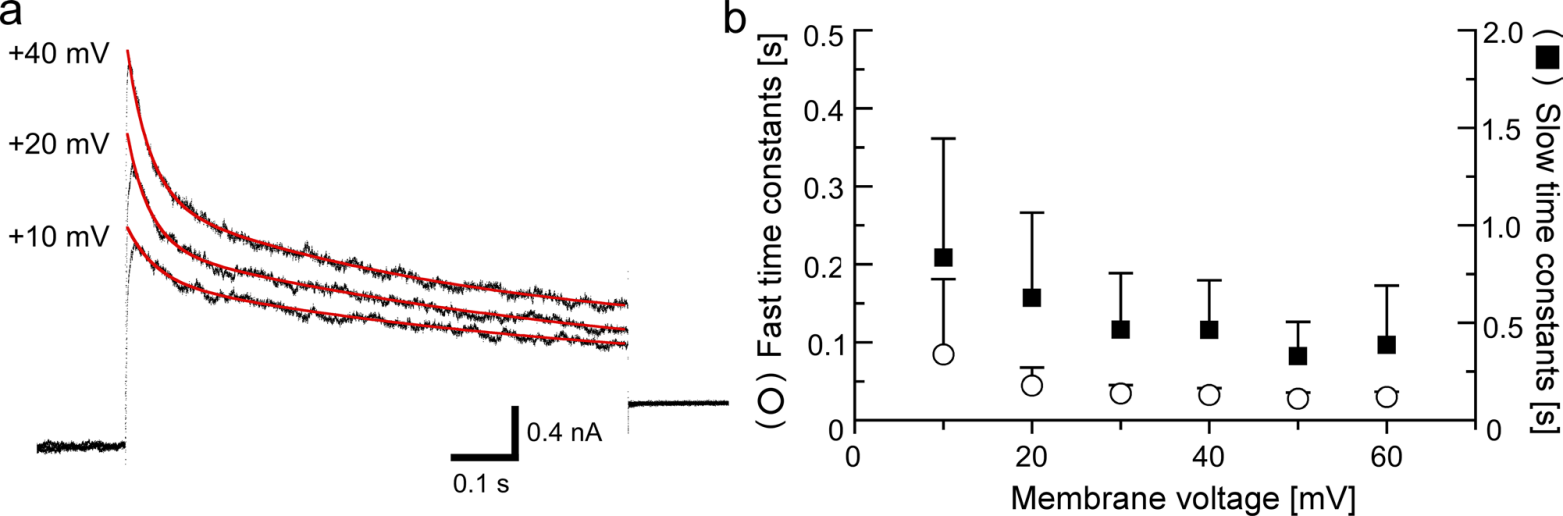
Inactivation time constants. Representative A-type K^+^ currents at the respective membrane potential of SNAP-25 immunoreactive type III cells (**a**). The currents traces were obtained by subtracting the current traces induced by activation plus from those by inactivation plus as shown in Fig. 3a. The inactivation processes were well fitted (red lines) with the double exponential equations. Voltage dependency of the inactivation time constants (**b**)

The results of the activation and steady-state inactivation suggest the functional expression of Kv3.3 and Kv3.4 channels in type III TBCs. The inactivation time constants of Kv3.4 is approximately one order faster than that of Kv3.3 currents, i.e., the inactivation time constants of Kv3.3 and Kv3.4 were 0.24 s and 10–20 ms at + 40 mV, respectively (Coetzee et al. 1999). Therefore, we examined the inactivation time constants of the A-type K^+^ currents in type III TBCs. The inactivation time constants were well fitted with double exponential equations (Fig. 4). The fast and slow time constants of type III cells were 32.3 ± 9.1 ms and 0.46 ± 0.25 s (n = 15) at +40 mV, respectively. These results suggest that both Kv3.3 and Kv3.4 channels functionally express type III cells.

### Recovery from inactivation

**Fig. 5:**
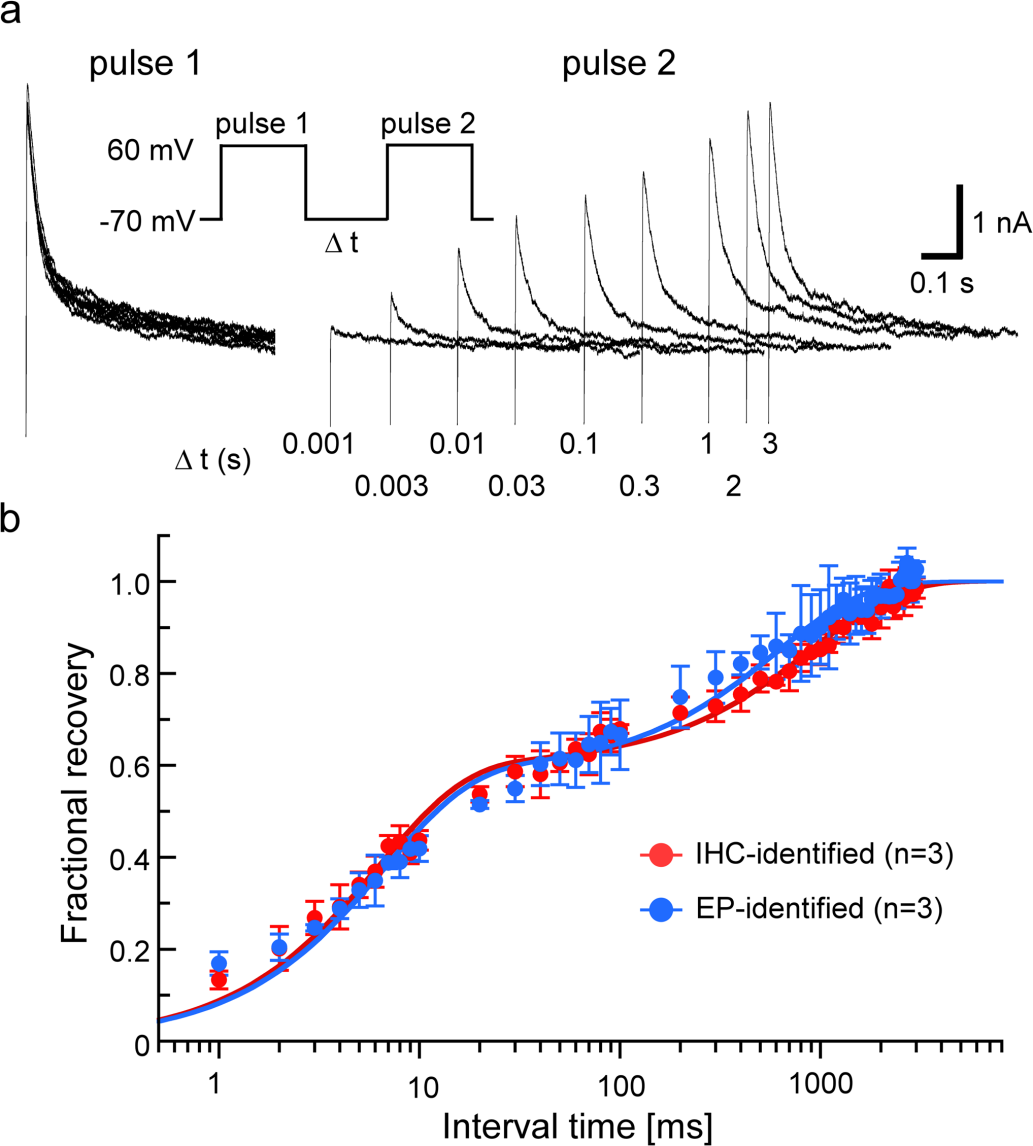
Recovery from inactivation. Representative A-type K^+^ currents in type III cells obtained using the double pulse protocol shown in the inset (**a**). Time course of recovery from inactivation of peak currents in immunohistochemically (IHC)-identified and electrophysiologically (EP)-identified type III cells (**b**). Fractional recovery was calculated by dividing the current magnitude during the test pulse (pulse 1) by the magnitude during the corresponding conditioning pulse (pulse 2). Recovery from inactivation was well fitted with double exponential (lines)

Figure 5a shows representative A-type K^+^ current traces obtained from type III cells. The A-type K^+^ currents recovered with increasing interval times and took approximately 3 s to recover completely. The time courses of recovery from the inactivation of the A-type K^+^ currents are shown in Fig. 5b. Recovery from the inactivation was well fitted with a double exponential equation. The fast time constants (τ1) of SNAP-25 immunoreactive and electrophysiologically identified type III cells were calculated as 6.3 ± 0.5 (n = 3, Fig. 5b and Table 2) and 6.5 ± 0.5 (n = 3) ms at − 70 mV, respectively, showing no significant differences (*P* = 0.36, unpaired t-test). The slow time constants (τ2) were 0.90 ± 0.07 s (n = 3) and 0.62 ± 0.32 s (n = 3) for SNAP-25 immunoreactive type III cells and electrophysiologically identified type III cells, respectively, showing no significant difference (*P* = 0.11, unpaired t-test). Furthermore, there was no significant difference in the fraction of the A-type K^+^ current described by τ1 between the SNAP-25 immunoreactive and electrophysiologically identified type III cells (*P* = 0.14, unpaired t-test, Table 2).

#### RT-PCR

**Fig. 6:**
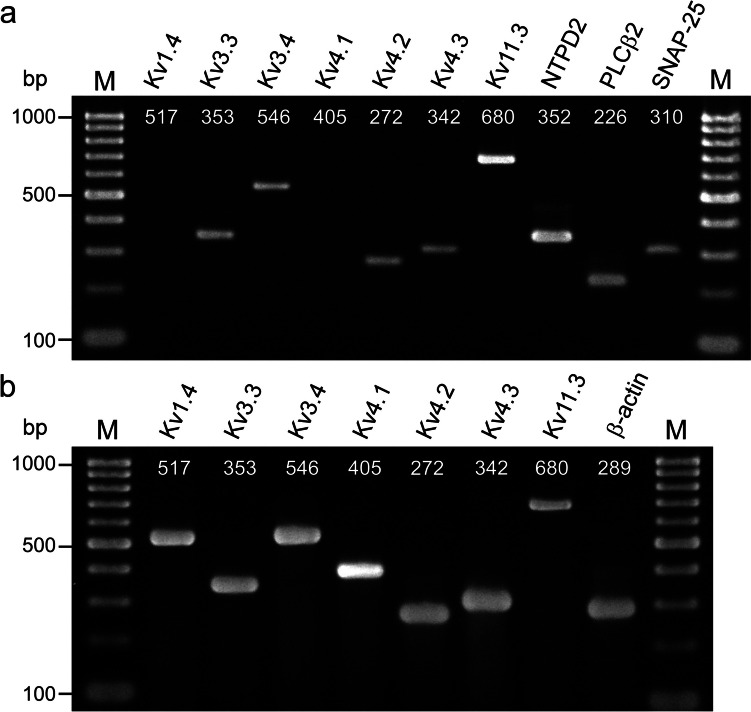
Expression of A-type voltage-gated K^+^ channel mRNAs in fungiform taste buds. Electrophoresis of RT-PCR products extracted from 30 taste buds (**a**). The numerals in each line indicate the expected product size amplified with each primer set shown in Table 1. NTPD2, PCLβ2, and SNAP-25 were used as positive controls for type I, II, and III cell markers, respectively. Positive controls (**b**), total RNA extracted from the mouse total brain was used. NTPD2, nucleoside triphosphate diphosphohydrolase-2; PCLβ2, phospholipase C β2; SNAP-25, synaptosomal-associated protein 25; bp, base pair; M, molecular weight marker

The electrophysiological feature of A-type K^+^ channels suggested the expression of Kv3.3 and Kv3.4 channels in type III cells. To confirm the gene expression of A-type K^+^ channels, we extracted total mRNAs from the mouse fungiform taste buds in the peeled lingue epithelia. Seven genes that exhibit rapid activating and inactivating K^+^ currents encoding six A-type K^+^ channels (Kv1.4, Kv3.3, Kv3.4, Kv4.1, Kv4.2, and Kv4.3) and Kv11.3 channels were examined by RT-PCR experiments. The results of the RT-PCR test demonstrated that five types of mRNAs, those for Kv3.3, Kv3.4, Kv4.2, Kv4.3, and Kv11.3, were detected using the extracted total RNAs from 30 taste buds (Fig. 6a). We performed seven individual experiments using extracted total RNAs from 20 or 30 taste buds. Kv3.3 mRNA was detected in all seven experiments. Among the seven experiments, Kv3.4 and Kv11.3 mRNAs were detected four times, whereas Kv4.2 and Kv4.3 mRNAs were detected three times. Kv1.4 and Kv4.1 mRNAs were not detected from the taste buds, although those mRNAs were detected by using total RNAs extracted from total mouse brain, as a positive control experiment (Fig. 6b). Although five types of mRNA were detected from fungiform taste buds, it is likely that Kv3.3 channels are predominant components in taste buds because the Kv3.3 mRNA was detectable with a high frequency and the electrophysiological features of A-type K^+^ currents were analogous to the Kv3.3 and Kv3.4 channels.

### Single-cell RT-PCR

**Fig. 7:**
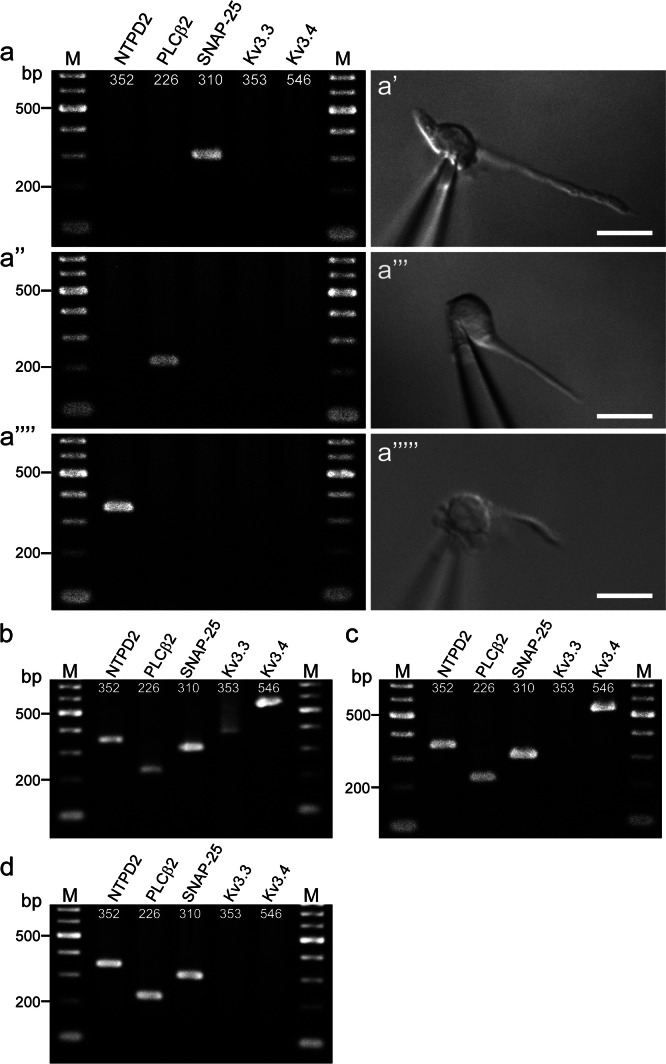
Single-cell RT-PCR experiments in fungiform taste bud cells. Electrophoresis of single cell RT-PCR (**a**, **a’’**, and **a’’’’**) and their respective images harvesting single TBCs (**a’**, **a’’’**, and **a’’’’’**). Numerals in each line indicate the expected product size amplified with each primer set. Scale bar in right panels, 10 μm. M, molecular weight marker. NTPD2, PCLβ2, and SNAP-25 were used as positive controls for type I, II, and III cell markers, respectively. Positive control of primer sets used for single-cell RT-PCR (**b**). Two taste buds were transferred into the first round RT-PCR tube. Positive control, one taste bud was transferred into the first round RT-PCR tube (**c**). Positive control, two taste buds were transferred into the first round RT-PCR tube (**d**). NTPD2, nucleoside triphosphate diphosphohydrolase-2; PCLβ2, phospholipase C β2; SNAP-25, synaptosomal-associated protein 25; bp, base pair; M, molecular weight marker

The electrophysiological features of A-type K^+^ currents in type III cells indicated the expression of high voltage-activated Kv3.3 and Kv3.4 channels. In addition, the RT-PCR experiments using total RNA samples extracted from 20 or 30 taste buds in fungiform papillae showed the expression of the Kv3.3 and Kv3.4 genes. These results suggest the expression of Kv3.3 and Kv3.4 mRNAs in type III cells. To confirm this, we performed single-cell RT-PCR experiments using a multiplex nested single-cell RT-PCR method (Ohtubo 2021). Unexpectedly, we could not detect the mRNAs of either the Kv3.3 or the Kv3.4 channels in type III cells (none of seven type III cells, Fig. 7a–a’). Moreover, as expected, neither mRNA of the Kv3.3 nor Kv3.4 channels was detected in type I (n = 20) or type II (n = 16) cells (Fig. 7a’’–a’’’’’). To verify the validity of the approach, we performed the experiments using 1–3 taste buds that were placed into a first-round RT-PCR tube. Only one out of nine samples expressed both the Kv3.3 and Kv3.4 mRNAs (Fig. 7b). Two samples expressed only the Kv3.4 mRNAs (Fig. 7c). In the case of the remaining samples, both the Kv3.3 and Kv3.4 mRNAs were undetectable, although cell-type marker genes could be detected (n = 6, Fig. 7d). These results suggested that although the present method could detect the both Kv3.3 and Kv3.4 mRNAs from a few taste buds, the expression level of these mRNA samples at a single cell might be below the detection limit, which was consistent with a previous study demonstrating that the expressions of Kv3.3 (Kcnc3) and Kv3.4 (Kcnc4) could hardly be recognized in type III cells (Sukumaran et al. 2017).

## Discussion

In the present study, we showed that the electrophysiological properties of A-type voltage-gated K^+^ currents in mouse fungiform TBCs and A-type K^+^ currents were expressed in type III taste receptor cells. Additionally, electrophysiological analysis of A-type K currents revealed the expression of Kv3.3 and Kv3.4 channels on type III cells. Moreover, the results of the RT-PCR experiments indicated that TBCs expressed the Kv3.3 and Kv3.4 genes, although both mRNAs remained undetected at the single-cell level. Hence, our data suggested that transient and high voltage-activated K^+^ channels, probably Kv3.3 and Kv3.4 channels, are the principal components of A-type K^+^ channels in type III fungiform TBCs. Since type III cells conduct sour taste information to the taste nerves and, in addition, form cell-to-cell communications among TBCs, the expression of A-type K^+^ channels on type III cells may affect the sour taste processing by neuromodulates released from the TBCs and efferent nerves.

The* Va*_1/2_ and *Vi*_1/2_ of A-type K^+^ currents in type III TBCs were 17.9 ± 4.5 (n = 17) and − 11.0 ± 5.7 (n = 17) mV, respectively (Fig. 3 and Table 2), which are analogous to the characteristics of Kv3 channels that exhibit more depolarized values, as compared to any other known voltage-gated K^+^ channels with at least 20–40 mV (Coetzee et al. 1999; Fernandez et al. 2003; Rudy and McBain 2001). Although the four Kv3 channels (Kv3.1–Kv3.4) have large voltage-dependent K^+^ currents with similar voltage dependency, Kv3.3 and Kv3.4 channels generate fast-activating and inactivating A-type K^+^ current with different inactivation time constants. The Kv3.3 currents inactivate slowly (time constant: 0.24 s at 40 mV), whereas the Kv3.4 currents inactivate relatively quickly (10–20 ms at + 40 mV) (Coetzee et al. 1999). Our data showed that the inactivation time constants of A-type K^+^ currents in type III TBCs [32.3 ± 9.1 ms and 0.46 ± 0.25 s (n = 15), respectively, at +40 mV] are relatively similar to those of both the Kv3.3 and Kv3.4 currents. Furthermore, RT-PCR experiments showed that, among the seven genes encoding A-type K^+^ channels, the Kv3.3 mRNA was detected in all seven individual experiments and Kv3.4 was detected four times. Previous studies showed that the main outwardly rectifying currents in type III cells were rapidly activating TEA- and Cs^+^-sensitive currents, i.e., voltage-gated K^+^ currents, whereas those in type II cells were mainly “slow activating TEA- and Cs^+^-insensitive currents” that probably passed through CALHM1/3 channels and hemichannels (Iwamoto et al. 2020; Kimura et al. 2014; Takeuchi et al. 2021). Taken together, it is likely that type III cells express Kv3.3 and Kv3.4 channels, although single-cell RT-PCR experiments did not detect both mRNAs. Moreover, because heteromultimeric Kv channels can be formed by the same subfamily, such as “Kv3.4 + Kv3.1,” that generates the A-type K^+^ currents (Rudy and McBain 2001; Weiser et al. 1994), further experiments are needed to investigate whether TBCs express Kv3.1 and Kv3.2 genes or not.

Electrophysiological experiments showed the expression of A-type K^+^ channel currents in type III cells, whereas single-cell RT-PCR experiments indicated that both the Kv3.3 and Kv3.4 mRNA remained undetectable in type III cells. This discrepancy might be explained by the differences between the mRNA and protein expression levels. For example, as reported in previous studies (Kimura et al. 2014; Takeuchi et al. 2021), all immunohistochemically identified type II and type III cells examined functionally generated voltage-gated Na^+^ currents (n = 15 and n = 9 cells, respectively). A single-cell RT-PCR experiment showed that 24% of type II cells (9/38) and 36% of type III cells (5/14) did not express any of the Nav1.3, Nav1.5, or Nav1.6 mRNAs, despite these cells expressing the type II or III cell type marker (Ohtubo 2021). In addition, four cells showed Nav mRNA expression without coexpression of cell type markers for type I, type II, or type III. Thus, the relationships between mRNA and protein expression levels are not simple. In general, it is known that there is not always a proportional relationship between mRNA and protein levels (Greenbaum et al. 2003; Washburn et al. 2003). Moreover, the expression of Kv3.3 and Kv3.4 mRNA has been found to be rare in type III cells (Sukumaran et al. 2017), which is consistent with our data demonstrating that at the single-cell level, both Kv3.3 and Kv3.4 mRNAs were at undetectable levels under our experimental conditions (Fig. 6a). However, at the taste bud level, both mRNAs could be detected using two different experimental methods (conventional RT-PCR using 20 or 30 taste buds and multiplex nested single-cell RT-PCR using 1–3 taste buds, respectively, Figs. 6 and 7). As a single taste bud in fungiform papillae contains two type III cells on average (Ohtubo and Yoshii 2011), we considered that mRNA levels reached the detection threshold by increasing the number of type III cells. Taken together, type III cells are likely to functionally express Kv3.3 and Kv3.4 channel proteins, but the corresponding mRNA levels remain below the detection limit in single type III cells. Furthermore, the possibility of cell cycle-dependent mRNA expression should not be excluded either (Buettner et al. 2015; Liu et al. 2016). As type III cells continuously turn over with a half-life of 22 days (Hamamichi et al. 2006; Perea-Martinez et al. 2013), Kv3.3 and Kv3.4 mRNA might be expressed at a specified period of cell cycle or at transcriptional bursting to stabilize protein levels over time. The samples might contain diverse states of cell cycles in type III cells by collecting a higher number of taste buds. Therefore, we could detect both the Kv3.3 and Kv3.4 mRNAs at the taste bud level. Further experiments would be required to clarify the relationship between Kv3.3 and Kv3.4 mRNA and their corresponding protein levels during the cell cycle of type III cells.

Kv3.3 and Kv3.4 channels contribute to the rapid repolarization of action potentials and action potential durations, enabling high-frequency firing (Coetzee et al. 1999; Kaczmarek and Zhang 2017; Rudy and McBain 2001). Nevertheless, the action potentials in type III TBCs responded to the sour tastant (10-mM HCl) were observed only at the rising phase of the membrane depolarization but not at the sustained plateau phase as shown in the present study (Fig. 1) and previous studies (Nakao et al. 2022; Ohtubo et al. 2001). The reasons for the difficulty of high-frequency firing in response to the sour tastants may be the slow recovery from the inactivation of A-type K channels, as indicated in the present study (Fig. 4), as well as the slow recovery from the inactivation of voltage-gated Na^+^ channels in type III cells (approximately 5 s for almost complete recovery) (Ohtubo 2021).

Otop1 proton channels are expressed on type III cells, and they play an important role in sour taste detection (Teng et al. 2019; Zhang et al. 2019). A proposed model for sour taste transduction indicates that intracellular acidification via protons occurring through Otop1 channels blocks Kir2.1 inward rectifier K^+^ channels, thereby inducing membrane depolarization and generating a train of action potentials (Teng et al. 2019). Additionally, only type III cells have conventional chemical synapses with taste nerve endings (Murray 1973; Seta and Toyoshima 1995), and they express soluble NSF attachment receptor proteins, such as SNAP-25 (Yang et al. 2000) and synaptobrevin-2 (Yang et al. 2004), as well as low and high voltage-activated Ca^2+^ channels (Clapp et al. 2006; DeFazio et al. 2006; Furue and Yoshii 1997). Therefore, the action potentials formed by Nav1.3 and Kv3.3 and Kv3.4 channels at the rising phase of the receptor potentials might be important for inducing the increment of intracellular Ca^2+^ concentrations, which initiate the vesicle-mediated synaptic transmission in type III cells.

The Kv3.3 and Kv3.4 channels expressed in the heterologous systems were modulated by protein kinase C (PKC) activators that suppressed the inactivation of both channels and enhanced the Kv3.3 current by several folds (Covarrubias et al. 1994; Desai et al. 2008). In small-diameter dorsal root ganglion nociceptors, the activation of PKC substantially slowed the Kv3.4 channel inactivation, narrowed the action potential waveform, and accolated the action potential repolarization, showing that the influence of Kv3.4 channel conductance on the action potential waveform was enhanced (Ritter et al. 2012). Additionally, the experiment using Kv3.3 knockout mice showed that the Kv3.3 channels control the neurotransmitter release at an excitatory synapse (Richardson et al. 2022). Since the action potentials in type III cells play a significant role in vesicle-mediated signal transduction, sour taste conduction may be affected by the phosphorylation of Kv3 channels that were regulated by the neurotransmitters secreted from the TBCs and efferent nerve endings. Furthermore, it was suggested that type III cells relay the taste information from type II cells to the taste nerves by forming the cell-to-cell communication in taste buds (Roper 2006; Roper and Chaudhari 2017). The phosphorylation of Kv3.3 and Kv3.4 channels in type III cells could modulate taste information of not only sour but also salty, sweet, bitter, and umami tastants within the taste buds. PKCε reportedly forms a complex with the Kv3.4 channel and primarily promotes its expression in a kinase activity-dependent manner (Zemel et al. 2021). The phosphorylation of Kv3.4 channels in type III cells may affect the expression levels of Kv3.4 channel, which could modulate taste information by changing the excitability of type III cells. In any case, the phosphorylation of Kv3.3 and Kv3.4 channels may affect the taste signal conduction via the type III cells. Further studies are needed to clarify the effect of phosphorylation on TBC excitability using a perforated whole-cell patch clamp that keeps intracellular small molecules, such as PKC, inside TBCs.

In conclusion, we have demonstrated that type III taste receptor cells in mouse fungiform taste buds expressed transient and high voltage-activated A-type K^+^ channels, probably Kv3.3 and Kv3.4 channels, based on the electrophysiological features. The results of RT-PCR experiments indicated that fungiform taste buds express Kv3.3 and Kv3.4 genes. As type III cells expressed TEA-sensitive delayed rectifying K^+^ channels in mouse fungiform taste buds, transient and high voltage-activated A-type K^+^ channels together with TEA-sensitive delayed rectifying K^+^ channels might contribute to the formation of the falling phase, especially the rapid repolarization, of action potentials. Given that TBCs form cell-to-cell communication, the phosphorylation of these channels via neuromodulators affect the synaptic transduction from type III to the taste nerves. Further experiments that take into account cell-to-cell communication are needed to clarify the taste signal modulation at the peripheral taste organs.

## Data Availability

The data underlying this article are available upon request to the corresponding author.

## Abbreviations

TBCs: Taste bud cells
Kv: Voltage-gated K^+^
SNAP-25: Synaptosomal-associating protein-25
IP_3_R3: Type 3 inositol 1, 4, 5-triphosphate receptor
